# Prognostic value of an immunohistochemical signature in patients with esophageal squamous cell carcinoma undergoing radical esophagectomy

**DOI:** 10.1002/1878-0261.12158

**Published:** 2018-01-06

**Authors:** Jin Meng, Junhua Zhang, Yingjie Xiu, Yan Jin, Jiaqing Xiang, Yongzhan Nie, Shen Fu, Kuaile Zhao

**Affiliations:** ^1^ Department of Radiation Oncology Fudan University Shanghai Cancer Center Shanghai China; ^2^ Department of Oncology Shanghai Medical College Fudan University Shanghai China; ^3^ Department of Pathology Fudan University Shanghai Cancer Center China; ^4^ Department of Thoracic Surgery Fudan University Shanghai Cancer Center China; ^5^ State Key Laboratory of Cancer Biology & Xijing Hospital of Digestive Diseases Fourth Military Medical University Xi'an China; ^6^ Department of Radiation Oncology Shanghai Proton and Heavy Ion Center Fudan University Cancer Hospital Shanghai China

**Keywords:** esophageal squamous cell carcinoma, immunohistochemical, least absolute shrinkage and selection operator model, prognostic, signature

## Abstract

Here, we aimed to identify an immunohistochemical (IHC)‐based classifier as a prognostic factor in patients with esophageal squamous cell carcinoma (ESCC). A cohort of 235 patients with ESCC undergoing radical esophagectomy (with complete clinical and pathological information) were enrolled in the study. Using the least absolute shrinkage and selection operator (LASSO) regression model, we extracted six IHC features associated with progression‐free survival (PFS) and then built a classifier in the discovery cohort (*n* = 141). The prognostic value of this classifier was further confirmed in the validation cohort (*n* = 94). Additionally, we developed a nomogram integrating the IHC‐based classifier to predict the PFS. We used the IHC‐based classifier to stratify patients into high‐ and low‐risk groups. In the discovery cohort, 5‐year PFS was 22.4% (95% CI: 0.14–0.36) for the high‐risk group and 43.3% (95% CI: 0.32–0.58) for the low‐risk group (*P* = 0.00064), and in the validation cohort, 5‐year PFS was 20.58% (95% CI: 0.12–0.36) for the high‐risk group and 36.43% (95% CI: 0.22–0.60) for the low‐risk group (*P* = 0.0082). Multivariable analysis demonstrated that the IHC‐based classifier was an independent prognostic factor for predicting PFS of patients with ESCC. We further developed a nomogram integrating the IHC‐based classifier and clinicopathological risk factors (gender, American Joint Committee on Cancer staging, and vascular invasion status) to predict the 3‐ and 5‐year PFS. The performance of the nomogram was evaluated and proved to be clinically useful. Our 6‐IHC marker‐based classifier is a reliable prognostic tool to facilitate the individual management of patients with ESCC after radical esophagectomy.

**Abbreviations**
AJCC: American Joint Committee on Cancer
AUROC: area under the receiver operating characteristic
DCA: decision curve analysis
EC: esophageal cancer
ESCC: esophageal squamous cell carcinoma
GST: glutathione *S*‐transferase
IHC: immunohistochemical
Ki LI: Ki67 labeling index
LASSO: least absolute shrinkage and selection operator
PFS: progression‐free survival
ROC: receiver operating characteristic

## 1 Introduction

Esophageal cancer (EC) is the sixth leading cause of cancer death worldwide, accounting for more than 400 000 deaths each year (Torre *et al*., 2015). EC consists of two types: squamous cell carcinoma and esophageal adenocarcinoma. In China, esophageal squamous cell carcinoma (ESCC) is the predominant histological type, which accounts for more than 90% of cases. Esophagectomy remains the mainstay of treatment for local ESCC. Multiple randomized clinical trials have shown a survival benefit with neoadjuvant or postoperative adjuvant treatment. Until recently, therapeutic approaches to EC were largely guided by American Joint Committee on Cancer (AJCC) staging system. Although significant advances in multimodality treatments have been achieved, the overall 5‐year survival rate for patients with EC remains variable (Law and Wong, 2002). Adjuvant treatment protocols are typically used in node‐positive patients after primary resection (Ando *et al*., 1997, 2003; Armanios *et al*., 2004; Macdonald *et al*., 2001; Pennathur and Luketich, 2008). However, clinicopathological risk factors might not be sufficient to distinguish the patients with high risk of disease progression. It is therefore important to identify biomarkers which may provide reliable prognostic information. Furthermore, prediction model integrating multiple biomarkers may enable clinicians to tailor the best combination of treatment, aiming at lowering disease mortality.

As an inexpensive and easy‐to‐use approach, immunohistochemical (IHC) assay is the most widely applied pathological technique in determining the expression of tumor‐associated proteins. IHC analysis is routinely used to differentiate between subtypes of EC. A panel of common markers have been used for the diagnostic of EC in clinical practice, including enzymes [TOPOII, glutathione *S*‐transferase (GST)‐π], oncogenes (c‐Myc, CyclinD1, EGFR, Her2/Neu), tumor‐specific antigens (MDR), tumor suppressor genes (p53, p21, p27), and tumor proliferation markers (Ki67, PCNA, BCL‐2, Bax). Growing evidence showed that IHC is a highly effective ancillary tool for predicting survival in patients with various cancer types. For example, a simple IHC panel with Ki67 and p53 has been reported for predicting patient outcome in luminal‐type breast cancer (Kobayashi *et al*., 2013). A three‐gene IHC panel has been reported to predict prognosis for patients with esophageal adenocarcinoma (Ong *et al*., 2013).

A number of IHC‐based biomarkers in predicting the prognosis of ESCC have been reported, but none have entered clinical practice (Shimada *et al*., 2002; Xu *et al*., 2016). Identification of prognostic models integrating multiple biomarkers may enable clinicians to tailor the best combination of treatment, aiming at lowering disease mortality.

The aim of this study was to develop and validate an IHC‐based classifier using the least absolute shrinkage and selection operator (LASSO) Cox regression model and establish a prognostic nomogram based on clinicopathological parameters and IHC biomarkers in a cohort of patients with ESCC after esophagectomy.

## 2 Methods

### 2.1 Patients and samples

Of the 324 patients who had undergone esophagectomy for locally resectable esophageal carcinoma at Fudan University Shanghai Cancer Center between 2007 and 2012, 235 patients with complete clinical and IHC information were enrolled in the study. This retrospective study was approved by the Institutional Review Board of Fudan University Shanghai Cancer Center and carried out in accordance with the Declaration of Helsinki. Written informed consents for tissue collection were obtained from all patients prior to inclusion. In this study, 67 patients with a pathological stage of T3–4 or N1–3 who had good performance status (Performance Status 0–1) have received postoperative concurrent chemoradiotherapy. Patients were further randomly stratified into discovery cohort (141 patients) and validation cohort (94 patients) as per 6 : 4 ratio.

### 2.2 Immunohistochemistry

All specimens analyzed were formalin‐fixed and paraffin‐embedded tissue sections. All the cases were reviewed by the junior pathologist and then independently reviewed by two practicing pathologists. Immunohistochemistry was performed on all cases included in this study. The IHC was performed by the Immunohistochemistry Diagnostic Laboratory of Fudan University Shanghai Cancer Center. The antibodies used, including EGFR (clone 5B7), Her2/Neu (clone 4B5), Ki‐67 (clone 30‐9), BCL‐2 (clone SP66), and CyclinD1 (clone SP4‐R), were all rabbit monoclonal (Ventana Medical Systems, Inc., , Tucson, AZ, USA). For these antibodies, tissue slides were processed using a BenchMark ULTRA automated immunostainer (Ventana). The antibodies used were mouse monoclonal (Shanghai Long Island Antibody Diagnostica, Inc., Shanghai, China), including anti‐c‐Myc antibody (clone 9E + 11), anti‐Bax antibody (clone G3‐31), anti‐p21 antibody (clone DCS‐60.2), anti‐P27 antibody (clone DCS‐72.F6), anti‐MDR antibody (clone P170), and anti‐GST antibody (clone 353‐10). The other antibodies, including PCNA (clone PC10), p53 (clone DO‐7), and TOPOII (clone 3F6), were mouse monoclonal (Leica Biosystems Newcastle, Ltd., Newcastle, UK).

Sections (4 μm thick) were cut from the formalin‐fixed, paraffin‐embedded blocks. Antigens were retrieved by microwave heating for 30 min in 10 mm sodium citrate (pH 6.0) for EGFR, Her‐2/Neu, c‐Myc, BCL‐2, MDR, GST‐π, and Bax or a Tris‐based buffer (pH 8.3) solution for 60 min at 95 °C for Ki67, p53, PCNA, p21, p27, CyclinD1, and TOPOII. To block endogenous peroxidase activity, the sections were treated for 5 min with 100% methanol containing 3% H_2_O_2_. The slides were incubated with primary antibodies at 4 °C overnight and then reacted with a dextran polymer reagent combined with secondary antibodies and peroxidase (EnVision Plus; Dako, Santa Clara, CA, USA) for 30 min at room temperature. And then, the sections were counterstained with Mayer's hematoxylin.

### 2.3 Evaluation of immunohistochemistry

PCNA, TOPOII, Bax, p53, p21, p27, CyclinD1, c‐Myc, and Ki‐67 IHC demonstrated consistent nuclei staining in tumor cells, while the markers including BCL‐2, MDR, and GST‐π were positive in the cytoplasm of the tumor cells. EGFR‐positive expression was observed on the membrane in tumor cells. The percentage of positive tumor cells and the maximum intensity of IHC signal (0–3) were recorded. Positive staining of the surface membrane, cytoplasm, and nucleus of tumor cells was noted and scored independently. Among them, the expression of Ki‐67 was assessed according to the percentage of positive staining cells found in 200 neoplastic cells (quantitative analysis), and Her‐2 IHC staining was scored according to the consensus panel recommendations for a gastric cancer scoring system (Park *et al*., 2012): IHC0 (negative) for no reactivity or < 10%; IHC1+ (negative) for faint/barely, part of membrane ≥ 10%; IHC2+ (equivocal) for weak to moderate, complete or basolateral ≥ 10%; and IHC3+ (positive) for moderate to strong, complete or basolateral ≥ 10%. All slides were evaluated independently by two pathologists who were blind to the clinical outcomes of the patients. (The expression of IHC markers is shown in Figs S1, S2, and S3.)

### 2.4 Development and validation of an immunohistochemical signature

The LASSO Cox regression method was chosen for the regression of high‐dimensional data. The most useful prognostic features were identified from the discovery cohort and were features with nonzero coefficients. The prognostic score of each patient was calculated via a linear combination of these features. A multimarker classifier was identified for predicting progression‐free survival (PFS) of patients with ESCC in the discovery data set. LASSO Cox regression model analysis was conducted by the ‘glmnet’ package using r software version 3.0.1 (R Foundation for Statistical Computing, Vienna, Austria).

### 2.5 Statistical analysis

We compared two groups using the *t*‐test for continuous variables and chi‐square test for categorical variables. Kaplan–Meier survival analysis and log‐rank test were used to estimate the survival time of patients in different risk groups stratified by IHC signature. The optimum cutoff point was selected using X‐tile plots based on the association with patients’ survival time. x‐tile software 3.6.1 (Yale University School of Medicine, New Haven, CT, USA) was used to assess the X‐tile analysis.

We investigated the prognostic performance of IHC signature using receiver operating characteristic (ROC) analysis. The ‘pROC’ package was applied to perform the ROC curve analysis. Univariable and multivariable Cox regression analyses were applied to analyze the independent prognostic effect of the signature. Cox regression coefficients were used to construct a nomogram for predicting the probability of PFS. Calibration plots were derived based on the regression analysis. We assessed the clinical utility of the nomogram by decision curve analysis (DCA). The nomogram and calibration plots were carried out using the ‘rms’ R package. Statistical analysis was performed with r software (version 3.0.1) and statistical levels were two‐sided, and statistical significance was set at 0.05.

## 3 Results

### 3.1 Clinical characteristics of patients

All patients in the cohort (235 patients) had undergone surgical resection, and 231 (98.3%) patients had histologically negative resection margins. The median follow‐up time was 30 months (range, 1–97 months), during which there were 149 relapses and 120 deaths. The clinical stage of the patients was determined based on the TNM classification according to AJCC 7th edition. Clinicopathological data were obtained from the medical records and pathology reports. Detailed clinicopathological characteristics of the discovery cohort (141 patients) and validation cohort (94 patients) are shown in Table 1.

**Table 1:** Pathoclinical characteristics of patients in discovery and validation cohort

	Training set		Validation set	
	Low‐risk patients (*n* = 73)	High‐risk patients (*n* = 68)	Low‐risk patients (*n* = 39)	High‐risk patients (*n* = 55)
Gender				
Male	63	63	33	52
Female	10	5	6	3
Age				
≥ 60	36	34	20	14
< 60	37	34	29	30
Tumor site				
Upper	17	25	6	15
Middle	39	26	20	25
Low	17	17	13	15
TNM stage				
IB (2)	14	6	9	6
IIA (3)	11	24	10	10
IIB (4)	21	7	6	13
IIIA (5)	19	16	6	16
IIIB (6)	4	8	4	6
IIIC (7)	4	7	4	4
Disease progression status				
No	36	18	19	12
Yes	37	50	20	43
Vascular invasion				
Absent	55	58	33	43
Present	18	10	6	12

### 3.2 Feature selection and immunohistochemical signature development

**Figure 1:**
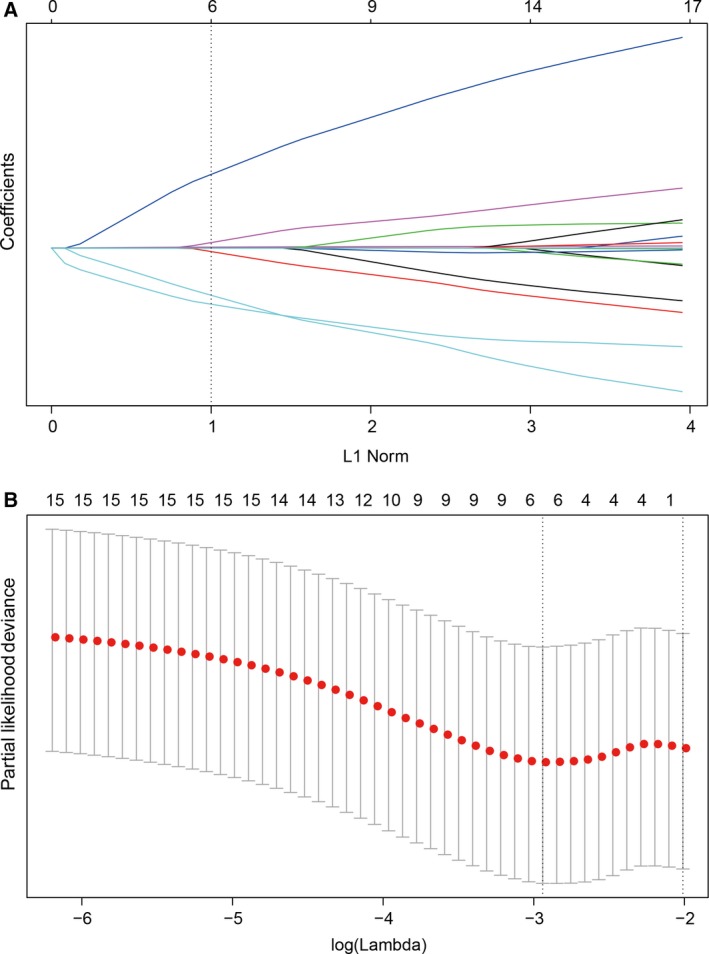
Feature selection using LASSO regression model. (A) Tuning parameter (selection by 10‐fold cross‐validation via minimum criteria. Partial likelihood deviance was plotted versus log(γ). (B) Coefficient profile of the IHC markers associated with PFS of patients with ESCC. Vertical line is shown at the optimal value with six nonzero coefficients.

**Figure 2:**
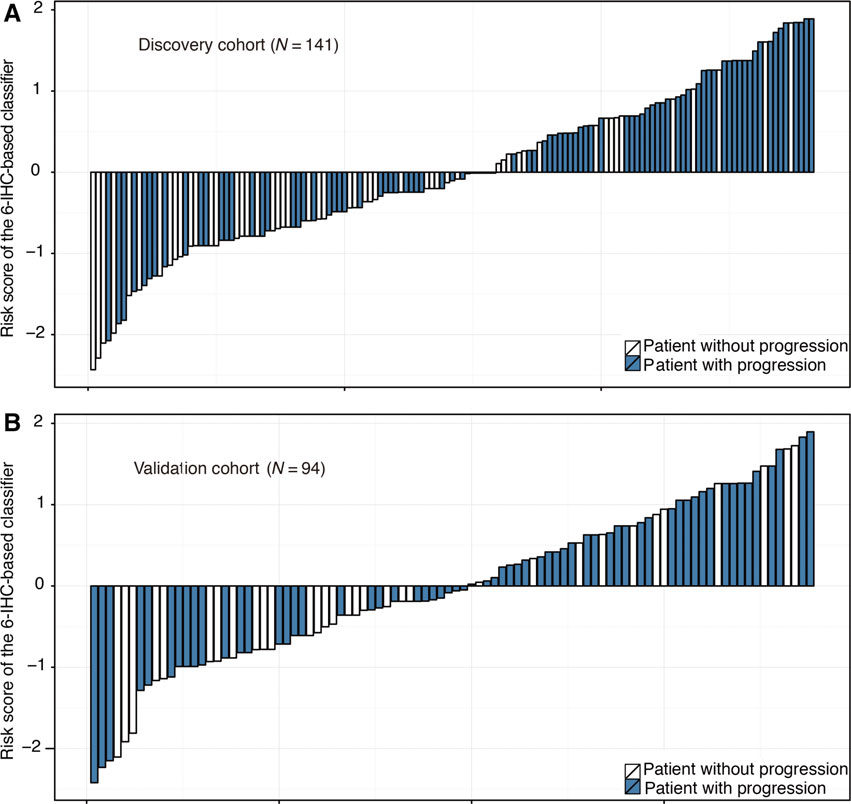
Distribution of risk score by 6‐IHC‐based classifier. (A) Discovery cohort and (B) validation cohort.

**Figure 3:**
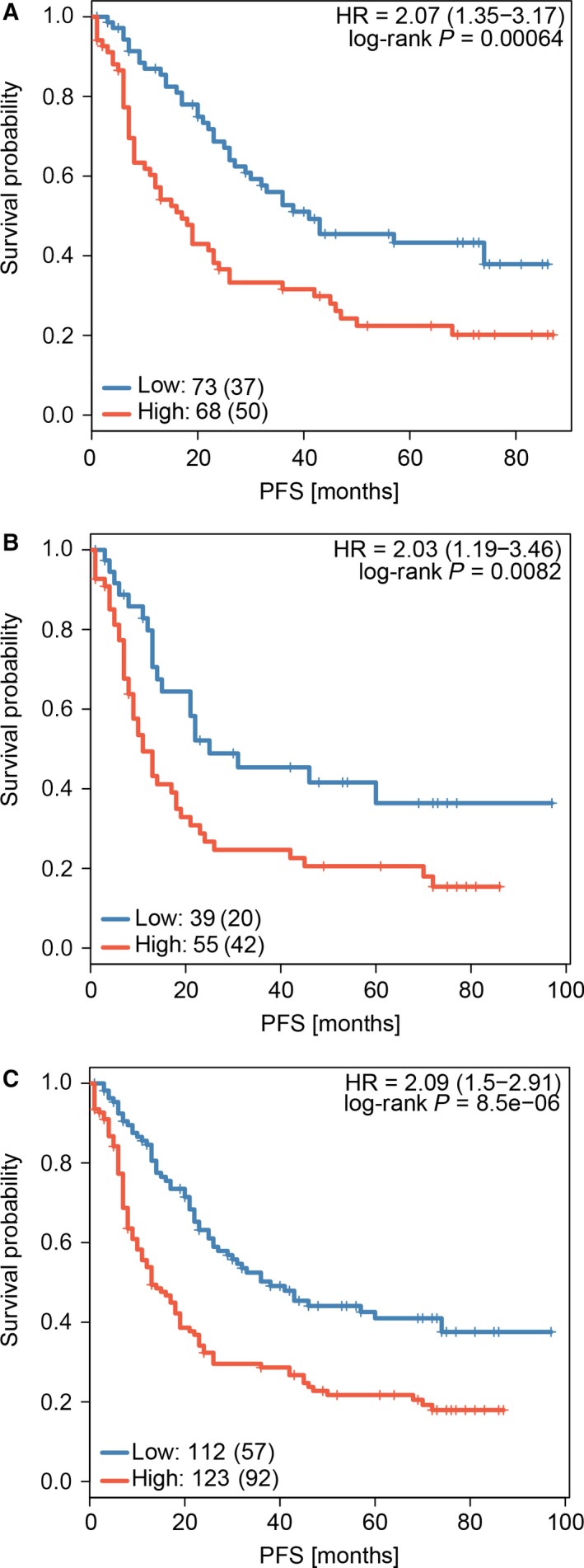
Comparison of PFS in high‐risk vs. low‐risk patients stratified by IHC signature. (A) Discovery cohort, (B) validation cohort, and (C) the combined cohort of discovery and validation groups.

We identified the potential predictive IHC markers using the LASSO Cox regression model. Of IHC markers (p21, p53, PCNA, c‐Myc, Neu, BCL‐2, Bax, Ki67, TOPO, MDR, GST‐π, p27, CyclinD1, and AgNOR), 14 features were reduced to six prognostic markers (p21, Her2/Neu, c‐Myc, Ki67, GST‐π, and p27) in the discovery cohort, and features with nonzero coefficients were enrolled in the regression model (Fig. 1). According to the expression status of the six IHC markers, we derived a formula to calculate the risk score of individual patient, based on their individual six prognostic marker expression levels: risk score = 0.044674285*p21 − 0.457229645* Her2/Neu + 0.325657944*c‐Myc + 0.005511644*Ki67 + 0.297544856*GST − 0.056460672*p27. In this formula, negative status of IHC equals 0 and positive status equals 1. The optimum cutoff level of six markers was defined as 0.56 by the X‐tile plot approach (Fig. S4). To simplify the clinical utility, an adjusted value (−0.56) was applied in the final formula (Fig. 2). Using this formula, we classified the patients in the discovery cohort into low‐ and high‐risk groups. Patients with a risk score of 0 or higher were included in the high‐risk group, whereas those with a risk score lower than 0 in the low‐risk group. Based on risk score, 141 patients of discovery cohort were further stratified into high‐risk group (68 patients, 48.2%) and low‐risk group (73 patients, 51.8%). Patients with lower risk scores have better 5‐year PFS. Five‐year PFS was 22.4% (95% CI: 0.14–0.36) in the high‐risk group and 43.3% (95% CI: 0.32–0.58) for the low‐risk group (*P* = 0.00064; Fig. 3A).

### 3.3 Validation of the signature

Patients stratified into different risk groups have significantly different survival. Patients with lower risk scores generally have longer PFS time than those with higher risk scores. The same analysis was carried out in the validation cohort (94 patients). Using risk score, we classified these patients into high‐risk group (55 patients, 58.5%) and low‐risk group (39 patients, 41.5%). Five‐year PFS was 20.58% (95% CI: 0.12–0.36) for the high‐risk group and 36.43% (95% CI: 0.22–0.60) for the low‐risk group (*P* = 0.0082; Fig. 3B). Similar differences between the two groups were noted in the combined training and validation cohort (*P* = 8.5e‐6; Fig. 3C).

### 3.4 Prediction accuracy of IHC signature

**Figure 4:**
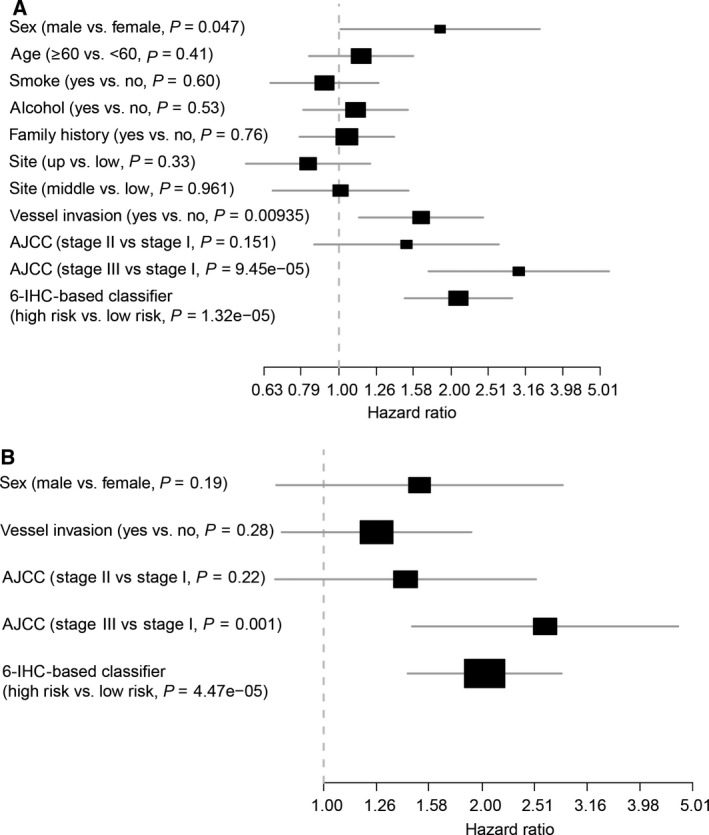
Analysis of clinicopathological information with PFS. (A) Univariate and (B) multivariate analysis of clinicopathological information with PFS

In univariable analysis, AJCC staging, 6‐IHC marker‐based classifier, gender, and vessel invasion status were found to be significant prognostic factors, while other clinicopathological factors showed no statistic differences (Fig. 4A). Multivariate analysis showed that only AJCC staging and IHC‐based classifier remained independent predictors for PFS (Fig. 4B).

**Figure 5:**
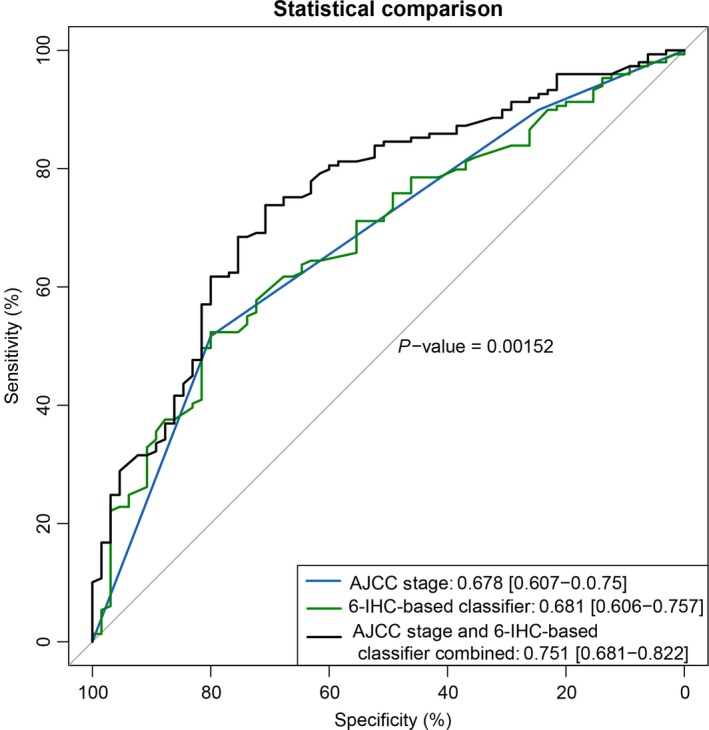
ROC curve analysis compares the prognostic value of IHC signature with AJCC staging.

Moreover, the time‐dependent ROC curve analysis indicated that the area under the receiver operating characteristic (AUROC) of the classifier was 0.751, which was similar to that of the AJCC TNM classification. Furthermore, the combination of 6‐IHC marker‐based prediction and AJCC‐based model had better performance for predicting PFS than the AJCC TNM classifications alone (*P* = 0.00152). Thus, the 6‐IHC marker‐based classifier could add prognostic value to AJCC stage in predicting the recurrence risk and survival (Fig. 5).

### 3.5 Nomogram building and its clinical utility

**Figure 6:**
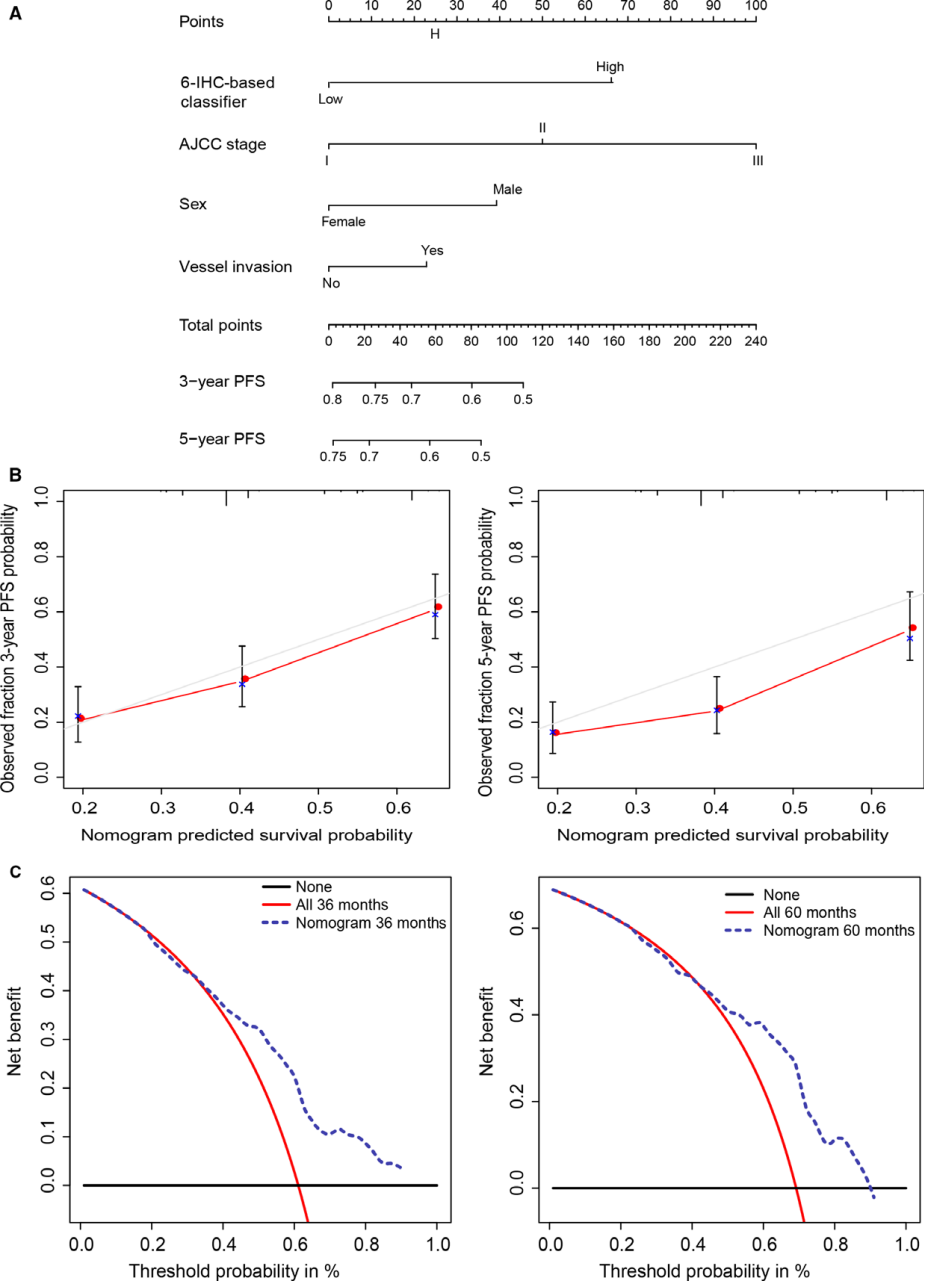
Nomogram (A) Nomogram integrating IHC markers and clinicopathological factors (B) Evaluation of nomogram using calibration curves: 3‐ and 5‐year nomogram calibration curves. The dashed line represents an ideal evaluation, whereas the red line represents the performance of the nomogram. (C) DCA to evaluate clinical utility of nomogram. The *y*‐axis measures the net benefit. The dashed line represents the nomogram. Using the IHC‐based nomogram to predict PFS could add more benefit than the treat‐all‐patients or the treat‐none‐patient strategy.

To provide a clinically useful tool to predict the prognostic, we constructed a nomogram integrating 6‐IHC markers and multiple clinicopathological risk factors associated with PFS. Gender, vessel invasion status, and AJCC staging were included in the prediction model (Fig. 6A). Calibration curves showed good performance of the nomogram with high consistency between the 3‐ or 5‐ year PFS estimates from the nomogram and those derived from Kaplan–Meier estimates.

Decision curve analysis was used to evaluate the potential of clinical application of the IHC‐based nomogram by quantifying the net benefits (Fig. 6B). The threshold probability at which a patient would opt for treatment informs us how a patient weighs the relative harms of false‐positive and false‐negative prediction. Here, the relative harm of treatment is equal to the expected benefit of avoiding treatment. This theoretical relationship is then used to derive the model which plots the net benefit against threshold probabilities. The net benefit was calculated by subtracting the proportion of all patients who are false‐positive from the proportion who are true‐positive. The nomogram demonstrated high potential of clinical application as better net benefits are ensured through the range of threshold probabilities for 3‐ or 5‐year PFS compared with the treat‐all or the treat‐none option (Fig. 6C).

## 4 Discussion

Substantial controversy exists regarding the appropriate indications for adjuvant therapy of patients with ESCC after esophagectomy (Pennathur *et al*., 2013). Despite many advantages, it is difficult and inaccurate to predict prognosis after potentially curative surgery for ESCC. In recent studies, multimarker assays incorporating individual markers into marker panel have been widely validated in various cancer types to predict the likelihood of recurrence and the benefit of adjuvant therapy (Birkhahn *et al*., 2007; Gorelik *et al*., 2005). However, current methods have not delivered clinically useful molecular prognostic biomarkers in ESCC. To help guide management decisions, we used a cohort of patients after esophagectomy to identify clinically useful IHC biomarkers and develop an IHC marker‐based nomogram to estimate 3‐ and 5‐year PFS among patients with ESCC after esophagectomy.

The LASSO Cox regression method was used for shrinkage of features and selection of best combination of outcome predictors. This is a regression analysis method which both performs the selection of predictors and combines the selected features to construct a model. LASSO Cox regression method has been applied to incorporate multimarker panels in recent studies, such as constructing radiomics nomogram for preoperative prediction of lymph node metastasis in colorectal cancer and building miRNA signature in stage II colon cancer (Huang *et al*., 2016; Zhang *et al*., 2013). In the current study, 14 features were reduced to six potential predictors on the basis of discovery cohort by shrinking the regression coefficients with the LASSO method. By incorporating six IHC items into a signature, patients were stratified into low‐risk and high‐risk groups. Patients in the low‐risk group have significantly better survival than those in the high‐risk group. Further, we validated the potential value of signature in predicting the prognosis among patients in the validation cohort. Multivariate analysis showed that the signature was an independent prognostic factor for PFS when adjusted by clinicopathological variables. Combined with AJCC staging, the classifier showed significantly better prediction of PFS than AJCC staging system alone. In addition, the IHC signature and the clinicopathological variables of poor prognostic features including gender and vessel invasion status and AJCC staging were integrated into a prognostic nomogram. Calibration plots revealed a good correlation between the predicted survival probability and the actual survival rate. The DCA showed high potential of clinical application of the nomogram.

Immunohistochemical analysis is a clinically practical tool in terms of availability and labor requirements, and at post‐transcriptional processing level. We constructed an IHC‐based nomogram which provides improved risk stratification and might be a prognostic tool for facilitating clinical management of treatment. The biological role of proteins in our panel has been previously reported. The expression of Ki67 is associated with cell proliferation status. The Ki67 protein is present during all active phases of the cell cycle (G1, S, G2, mitosis) except for the resting phase (G0) (Bullwinkel *et al*., 2006). The fraction of Ki67‐positive tumor cells (Ki67 labeling index, Ki LI) has been proven to be an established prognostic marker for various tumor types, especially in breast cancer (Hu *et al*., 2017; Li *et al*., 2017). Previous studies have shown a strong linear correlation between Ki‐67 labeling index and esophageal adenocarcinoma development (McCormick Matthews *et al*., 2015). However, the prognostic significance of Ki67 expression in ESCC remains inconclusive. The p21 gene plays an important role in cell cycle regulation by inhibiting the activities of cyclin/cyclin‐dependent kinase (CDK) complex (Lukas *et al*., 1996). The expression of p21 protein is regulated by wild‐type p53. The relationship between p21 expression and ESCC has been investigated, whereas the prognostic role of p21 remains controversial. It has been reported that p21 could serve as a positive prognostic predictor for patients with ESCC (Liu *et al*., 2012; Shiozaki *et al*., 2013). The pattern of p21 and p53 expression might predict a favorable prognosis of patients with advanced ESCC (Natsugoe *et al*., 1999). However, some studies obtained contrasting results (Goan *et al*., 2005; Taghavi *et al*., 2010). As an important β‐catenin target gene, c‐Myc is involved in growth control and proliferation of cells. The expression of c‐Myc has been immunohistochemically evaluated and found to be associated with the phenotype of ESCC (Wang *et al*., 2011). GST π is one of the isoforms identified in GST family. Numerous studies have suggested that GST‐π is a marker protein for the detection of chemical toxicity and carcinogenesis (Aliya *et al*., 2003; Townsend and Tew, 2003). GST‐π plays an important role in regulating the MAP kinase pathway via protein–protein interactions. The expression of GST‐π protein has been reported to be correlated with the prognosis in human esophageal squamous carcinoma (Ishioka *et al*., 1991; Wang *et al*., 2010). The CDK inhibitor p27 protein exerts both positive and negative functions on cell proliferation, cell motility, and apoptosis regulation. p27 expression level may serve as a prognostic and therapeutic implication biomarker in various cancer types. The clinical importance of amplification of Her‐2/Neu (c‐erbB‐2) has been proved in breast cancer. Numerous studies found that either HER2 gene amplification or protein expression was a predictor for unfavorable prognosis in breast cancer. The rates of HER‐2 gene amplification have been evaluated in ESCC and found to be less than gastroesophageal junction and gastric adenocarcinoma (Huang *et al*., 2013). In this study, we succeeded in integrating multiple IHC markers into one model by applying the LASSO Cox regression model, which has significantly greater prognostic accuracy than that of single IHC marker alone (Fig. S5).

Although the IHC marker‐based nomogram demonstrated good predictive accuracy for survival of patients with ESCC patients, our current study has several limitations. First, the nomogram was established based on retrospective data from an individual cancer center. Second, our study lacks genomic characteristics for the validation of biomarkers. Third, further prospective study in multicenter clinical trials will be required to further validate our results.

In conclusion, we developed and validated a nomogram integrating IHC markers and clinicopathological characteristics, which can be performed to accurately predict the prognosis of patients with ESCC after radical esophagectomy. Predicting survival of patients with accurate prognostic models would be greatly beneficial for selection of optimal therapeutic strategies and individualized patient counseling.

## Author contributions

KZ conceived the idea. JM and JZ designed the experiments, analyzed the data, and wrote the manuscript. JM, JZ, and JX performed the experiments. YX and YJ helped in interpreting the pathological results. YN, KZ, and SF critically revised the manuscript. All authors approved the final version.

## Supporting information


**Fig. S1.** (A–C) Squamous cell carcinoma of different grades. (H&E. magnification ×40).

**Fig. S2.** (A–D) p21, p53, and c‐Myc were strongly positive in nuclei of tumor cells; GST was diffusely positive in the cytoplasm of tumor cells (EnVision, DAB, magnification ×200), (E–H) while the expression of those markers above was lost in part of the cases (EnVision, DAB, magnification ×200).

**Fig. S3.** (A–C) HER2 is positive on the membrane of tumor cells. A: 1 + ; B: 2 + ; C: 3 +  (EnVision, DAB, A: magnification ×200; B and C: magnification ×400). (D–F) Ki‐67 index were measured ~ 30%‐85%, respectively. D: 30%; E: 50%; F: 85% (EnVision, DAB, magnification ×200).

**Fig. S4**. X‐tile analysis was performed using training set.

**Fig. S5**. ROC curve analysis of each IHC marker.
