# Supplementary figures and images for: Prognostic value of an immunohistochemical signature in patients with esophageal squamous cell carcinoma undergoing radical esophagectomy
Source: Mol Oncol. 2018 Jan 6;12(2):196–207. doi: 10.1002/1878-0261.12158 (PMC5792740; doi:10.1002/1878-0261.12158)

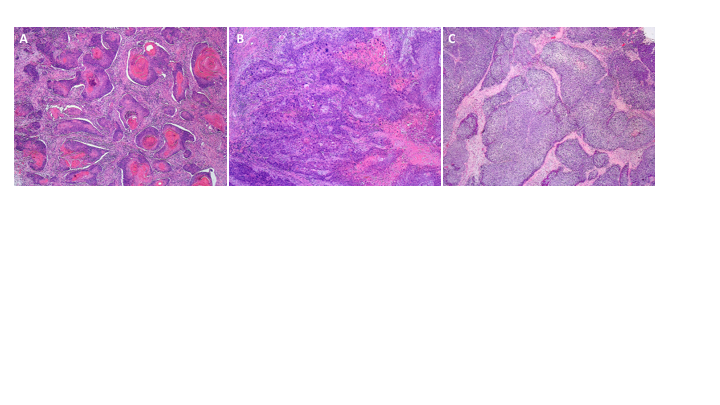

Supplement: Supplementary file 1 — Fig. S1. (A–C) Squamous cell carcinoma of different grades. (H&E. magnification ×40). [file MOL2-12-196-s001.tiff]

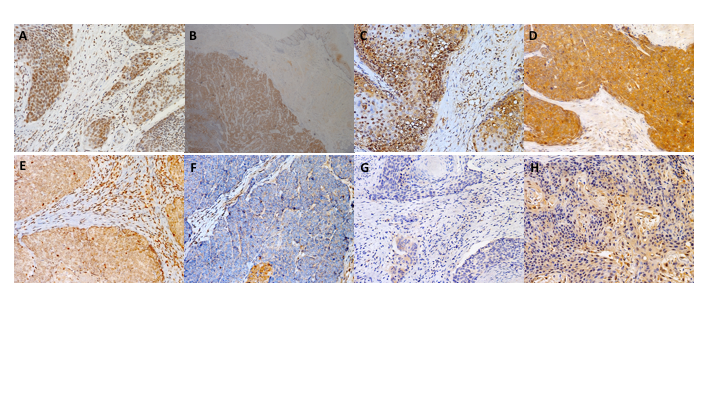

Supplement: Supplementary file 2 — Fig. S2. (A–D) p21, p53, and c‐Myc were strongly positive in nuclei of tumor cells; GST was diffusely positive in the cytoplasm of tumor cells (EnVision, DAB, magnification ×200), (E–H) while the expression of those markers above was lost in part of the cases (EnVision, DAB, magnification ×200). [file MOL2-12-196-s002.tiff]

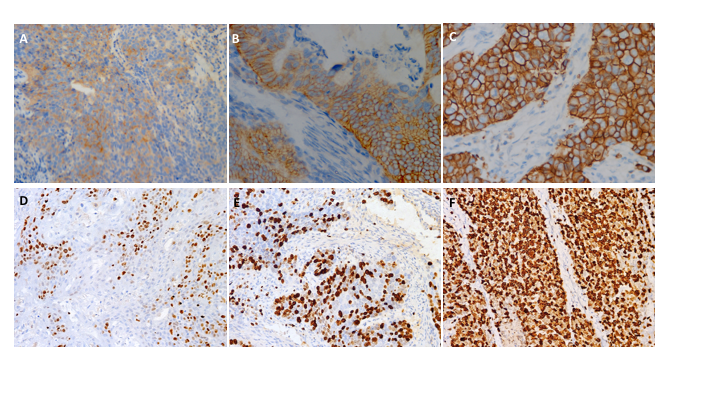

Supplement: Supplementary file 3 — Fig. S3. (A–C) HER2 is positive on the membrane of tumor cells. A: 1 + ; B: 2 + ; C: 3 + (EnVision, DAB, A: magnification ×200; B and C: magnification ×400). (D–F) Ki‐67 index were measured ~ 30%‐85%, respectively. D: 30%; E: 50%; F: 85% (EnVision, DAB, magnification ×200). [file MOL2-12-196-s003.tiff]

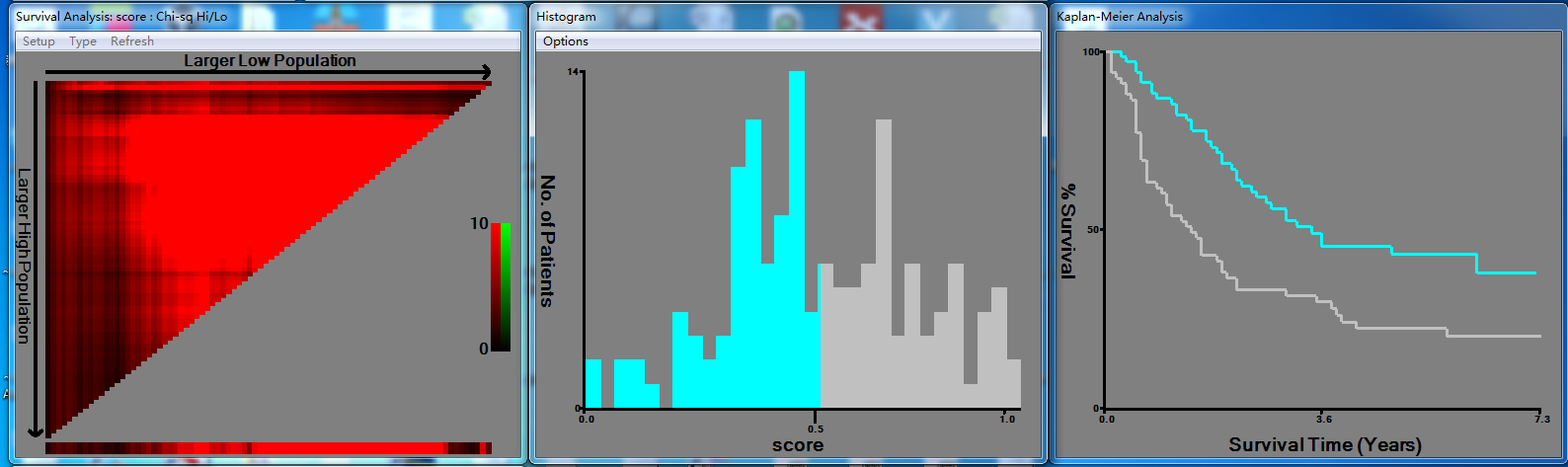

Supplement: Supplementary file 4 — Fig. S4. X‐tile analysis was performed using training set. [file MOL2-12-196-s004.tif]

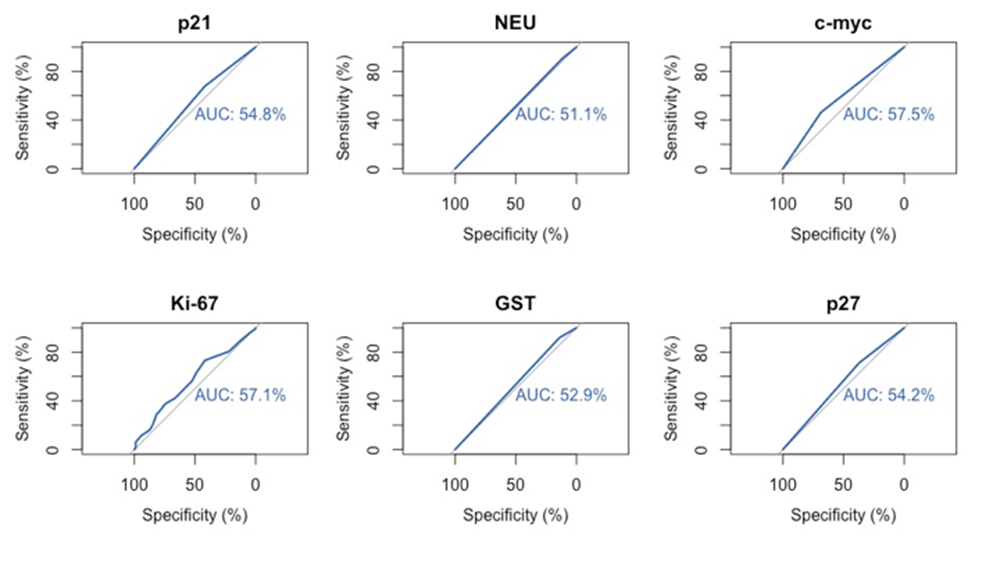

Supplement: Supplementary file 5 — Fig. S5. ROC curve analysis of each IHC marker. [file MOL2-12-196-s005.tif]
